# Results of a Codesign Process: A Cognition Screening Pathway for Inpatient and Outpatient Settings for Patients Who Are Facing or Have Undergone Lower Limb Amputation

**DOI:** 10.3390/jcm13237378

**Published:** 2024-12-04

**Authors:** Erinn Dawes, Lyndel Hewitt, Vida Bliokas, Val Wilson

**Affiliations:** 1 School of Nursing, Faculty of Science, Medicine and Health, University of Wollongong, Wollongong, NSW 2522, Australia; 2 Illawarra Shoalhaven Local health District, Illawarra Transitional Aged Care Service, Block E Port Kembla Hospital, Warrawong, NSW 2502, Australia; 3 Faculty of Science, Medicine and Health, University of Wollongong, Wollongong, NSW 2522, Australia; lhewitt@uow.edu.au; 4 School of Psychology, Faculty of Arts, Social Sciences and Humanities, University of Wollongong, Wollongong, NSW 2522, Australia; vida@uow.edu.au; 5 South West Sydney Nursing and Midwifery Research Alliance, The Ingham Institute, 1 Cambell Street, Liverpool, NSW 2170, Australia; valerie.wilson@health.nsw.gov.au

**Keywords:** codesign, cognition, cognitive screening, amputation, multidisciplinary

## Abstract

**Background/Objectives**: Cognition plays a major role in prosthetic rehabilitation success. The ability to identify patients who may have difficulty understanding and adapting to the rehabilitation process is beneficial for clinicians and patients to allow for targeted and appropriate therapy. The research aim was to codesign a process that facilitates routine cognitive screening into the amputee inpatient journey. **Methods**: A convenience sample of sixteen medical and allied health practitioners from one local health district undertook a codesign process over 10 months from March to November 2023. A combination of virtual and face-to-face data collection occurred. Each of the codesign meetings was audio recorded, following which transcription occurred. Transcripts were reviewed using thematic analysis-based techniques to capture themes and consensus within the group. **Results**: Two pathways were established for use within one local health district, allowing clinicians to measure the cognition of patients in both inpatient and outpatient settings either before or after they underwent amputation. The newly established pathways provide step-by-step guidance for clinicians, such as how to address contraindicators for testing and providing guidance for subsequent neuropsychological testing. The Montreal Cognitive Assessment (MoCA), both paper based and electronic based, was selected as the cognitive screening tool for implementation. **Conclusions**: Utilizing codesign as a method for generating a cognitive screening pathway for amputees was successful. The pathways generated should be reviewed for suitability for application in other health settings.

## 1. Introduction

Within a healthcare environment, intact cognition is assumed for most patients; however, some may experience a decline in their cognitive function for a plethora of reasons including, but not limited to, the onset of delirium [1] as a result of a medical event such as a stroke [2] or an infection [3]. These changes can represent challenges for both the patient and the treating team, particularly around the patient’s insight into their condition(s), their ability to make decisions about their care, and having the capacity to consent to procedures [4]. Determining if a patient retains the ability to make decisions about their care is made more difficult by the fact that neither neuropsychology nor neurology have been able to reach a definitive definition of what abnormal cognition is [5]. Abnormal cognition may come in the form of mild cognitive impairment (MCI) where there are changes in one area of cognition without impairing the patient’s ability to complete activities of daily living [6]. Alternatively, it may come in the form of vascular mild cognitive impairment (VMCI) where patients experience changes in processing speeds and executive function while retaining episodic memory [7,8]. With each abnormal cognition presenting differently, there are several ways (screening tools and assessment measures) that hospital staff seek to determine if a patient has the cognitive capacity to make decisions pertaining to their care and health.

The most commonly used cognitive screen in clinical practice is the Mini-Mental State Examination (MMSE) [9]. Other brief cognitive screens include the Montreal Cognitive Assessment (MoCA), the Cambridge Cognitive Examination (CAMCOG), and the Addenbrooke’s Cognitive Examination (ACE) [9,10]. The primary purpose of a cognitive screening tool is to measure a range of cognitive functions in a brief manner that is sensitive and specific enough to provide early identification of individuals at potential risk for a condition that would be meaningful beyond what could be associated with aging [9,10]. A secondary consideration, usually of equal importance for clinicians, is that the cognitive screening measures are easy to use. If necessary, more in-depth cognitive assessments are completed by a neuropsychologist when available and can provide a greater degree of accuracy and detail about a patient’s cognitive function, as well as determine the functional limitations a patient may report [10,11].

There are several ways in which a person may lose a lower limb. Congenital limb differences, trauma, and subsequent health conditions (e.g., cancer or diabetes) are three such ways [12,13,14]. Amputation due to diabetes has been gaining attention as there is increased realization that diabetes as a co-morbidity may impact on more than just vascular limb loss pathways. Research by Cheng et al. found that having diabetes predisposed patients to Alzheimer’s disease and MCI, with Palta et al. later building on that and identifying that the cognitive profile of a diabetic demonstrated changes to verbal and visual memory, processing speed, executive function, and motor performance [15,16]. Later, Lombard-Vance found that all patients who underwent amputation, compared with normative data, had lower overall cognitive functioning, with particular deficits in reasoning, information processing, visuospatial function, and executive function [17].

The overlap between diabetic cognitive changes and cognitive changes associated with amputation (all cause) highlight the importance of cognition when considering prosthetic rehabilitation. Prosthetic rehabilitation requires a patient to redevelop muscle memory for everyday tasks, such as walking. Previously, these tasks would have been subconscious; however, success post amputation is dependent upon neural plasticity in response to changed neural pathways [18]. There is a cognitive burden associated with understanding and applying new meaning to signals sent from areas of reduced proprioception while learning new motor tasks, and this can make prosthetic rehabilitation, and indeed mobility, challenging [18,19].

Three following factors drove the direction of this research:

- Understanding that a cognitive burden accompanies prosthetic rehabilitation.
- Knowledge that a leading local agency responsible for the innovation in clinical healthcare for the state of New South Wales (NSW) advocated for all post-operative amputation patients to have routine cognitive screening [20].
- Knowledge that the health district under investigation was falling short of the recommendations to meet cognitive screening [21].

Thus, the aim of this phase of the research was to codesign a process that facilitated routine cognitive screening as part of the amputee inpatient journey.

## 2. Materials and Methods

This study represents one phase of a larger action research study being undertaken by the lead author (ED) in a local health district within NSW, Australia. Building off the findings of earlier phases of the research (results of which can be found in [22]), a codesign method was used to assist in achieving the study aim, a process that ran over a ten-month period in 2023. Codesign is the process of bringing together participants or stakeholders who have local knowledge pertaining to the problem under investigation [23]. Those same participants are then co-leaders in the workable solution that is generated in response to the investigation [23]. The codesign methodology is detailed elsewhere [24].

### 2.1. Ethics

This research received approval from the joint University of Wollongong and Illawarra Shoalhaven Local Health District Medical Human research ethics committee (2021/ETH00462).

### 2.2. Participants

Due to the specialized nature of the area of inquiry and the focus on the local health district, a convenience sample of clinicians was used for the codesign process. Clinicians who have acute working knowledge of lower limb amputation (vascular surgeons and podiatrists), are part of the rehabilitation team (physiotherapists, occupational therapists, rehabilitation medicine physicians), and/or have knowledge of cognition and its assessment (psychologists) were identified as part of the convenience sample. Table 1 shows the inclusion and exclusion criteria for the codesign participants.

Participants were invited to participate through various channels, including presentation of previous data from an earlier phase, when their respective heads of department disseminated the invitation via email, or through word-of-mouth referral from a clinician colleague already participating in the codesign process. Clinicians who expressed an interest and agreed to participate were provided with a participant information sheet and consent form prior to commencement of codesign engagement. As the codesign meetings were monthly, the facilitator used a verbal consent agreement at the commencement of each meeting to reconfirm each participant’s agreement to participate.

It is important to acknowledge that the lead author (ED) is a clinician within the health district in which the research was completed. However, as the facilitator of the codesign process, there was no participation in the discussions or process of undertaking the codesign.

### 2.3. Data Collection

Data were collected over a ten-month period (March–November 2023) using virtual (Microsoft teams) and face-to-face means, led by the facilitator (ED). Each codesign meeting was between 60 and 90 min in length.

The initial codesign meeting provided an overview of the research undertaken in the district prior (results have been published elsewhere [21,22]) and utilized a topic guide to stimulate conversation. Topics included for discussion were those that had come out of prior phases of the research, including what cognitive screening tool should be used and when should the tool be used. Subsequent codesign meetings were more ad hoc in nature, building from the previous meeting in the construction of suitable screening pathways.

In order for all codesign participants to have a clear frame of reference while undertaking the codesign process, the initial codesign meeting saw the establishment of a way of working agreement, as well as a glossary of agreed definitions.

In each instance, the codesign meeting was recorded using the virtual online platform and a secondary audio recorder. Data were also collected in the form of a reflexive journal that the facilitator (ED) kept throughout the course of the ten months. A reflexive journal has been identified as a staple for qualitative researchers who need to be aware of their own biases and how they may be influencing the data. This also demonstrates the trustworthiness of the data that are being collected through reflexive capturing of questions and decisions being made as the study is taking place [25].

### 2.4. Data Analysis

Each of the codesign meeting recordings was transcribed to confirm accuracy of the facilitators’ recall of data and sequence of events. Thematic analysis-based techniques were used to review the themes found within the transcripts and ensure that consensus had been reached for each topic discussed. Furthermore, a combination of the transcripts and thematic analysis-based review assisted in the generation of a summary page that was disseminated to the participants following each meeting. The purpose of the summary page was to confirm progress and identify areas that were still under discussion. Transcription also allowed the data to be de-identified, with participants given pseudonyms for confidential reporting.

As the established aim was to create a pathway for healthcare service delivery design, data saturation was deemed to have occurred when all aspects of the pathway design had been explored and consensus gained with the codesign participants. In this instance, consensus was defined as an agreement that the proposed option best meets the needs and/or concerns of the majority of participants, and thus their represented disciplines. The definition of consensus was one that was established in the initial codesign meeting glossary, and having a clear understanding of how consensus was determined allowed the participants to define a shared goal [26].

## 3. Results

Five codesign meetings were run (four during the development phase and one follow-up meeting) across a ten-month period from March 2023 until November 2023. The number of participants at the meetings ranged from six to sixteen. The participants were five physiotherapists, four occupational therapists, three vascular surgeons, two rehabilitation medicine physicians, one psychologist, and one podiatrist. Disciplines that had larger staff numbers employed within the local health district also had greater numbers within the participant groups, making the codesign participant cohort representative of the different groups. Clinicians involved in the codesign process had two or more years of experience within their discipline, contributing a wide range of knowledge and experience to the codesign process.

There was consensus amongst the codesign group that cognition and its assessment within the amputee population was an important consideration that contributed to effective practice; this is summed up by Octavia who stated the following:

“I think there needs to be early cognitive assessment because I think part of the decision making process, informed consent and health literacy around knowing whether or not an amputation is right for you. Like is this person capable of making that decision”.

There was a lengthy and robust discussion regarding the best way to assess cognition. Consideration was on having a tool that targeted executive function as this would be implicated in a patient with a vascular profile, that it should not be too lengthy to complete, and having a tool that did not place a large burden of care on one particular discipline, with one participant, Poppy, remarking “Surely we just need something that everyone can do so that its inclusive”.

Several screening tools were identified and discussed for their utility in screening for cognition within the vascular amputee population. Table 2 lists the screening tools and their reasons for inclusion or exclusion within the pathway.

The final consensus between participants was that cognitive screening using the MoCA was the most appropriate, as it was a short screening tool that could be administered by a variety of disciplines if the electronic version (eMoCA) was utilized. Furthermore, the MoCA contained some elements that targeted executive functions that were identified as likely areas of deficit within the vascular amputee population. Consensus deemed that the RUDAS be used as a secondary screening tool for patients who did not have English as their primary language. Following determination of which tool to use, there was discussion around the timing of the screening tool being applied and what, if any, contraindicators would be in place to confirm the appropriateness of cognitive screening moving forward. There was also discussion and consensus reached on what further referrals would be appropriate if cognitive screening revealed changes that clinicians thought warranted further investigation.

**Figure 1:**
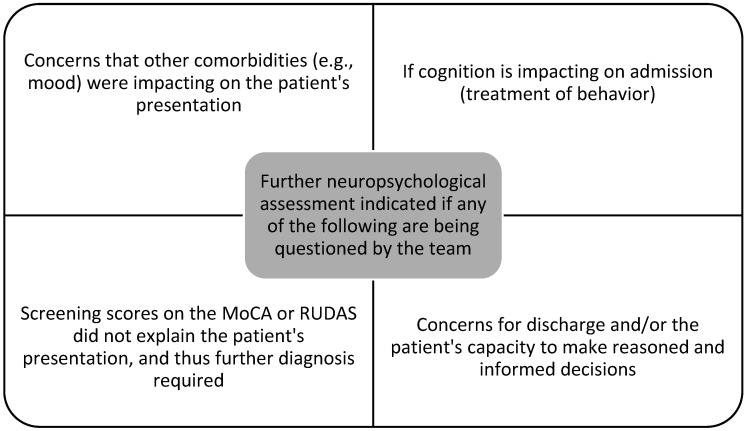
Criteria for neuropsychological examination following cognitive screen.

The codesign participants determined that, ideally, the screening tool would be completed on two occasions, the first prior to hospital admission via the high-risk foot clinic and podiatry and the second on admission to rehabilitation or transfer from an acute hospital ward, as this would capture the most patients, giving clinicians the best chance of having a baseline cognitive score available for comparison. There were three contraindicators for completing the cognitive screening that the codesign group established: a non-consenting patient, acute delirium, and acute or changed pain reporting. Delirium could be confirmed using the Confusion Assessment Method (CAM) [27] or the Delirium Risk Assessment Tool (DRAT) [28]. A criterion was established for subsequent referral to neuropsychology for further investigation, with these reasons being seen in Figure 1.

Noteworthy discussion on the topic of cognition centered around the decision to not use the final score of the cognitive screen in isolation. Clinicians were wary that documenting the cognitive screening scores into a patient’s medical record may lead to others using it as a reason to exclude from prosthetic casting, and thus the opportunity to undergo prosthetic rehabilitation. One participant, Olivia, noted “if we did the screen and there were concerns, we would do something functional. Get them in the kitchen or something like that to see if it kind of correlates to functional tasks”. To address this concern, the participants agreed that with documentation of the cognitive screening score there would be a statement that “This score needs to be taken in conjunction with a functional assessment”.

**Figure 2:**
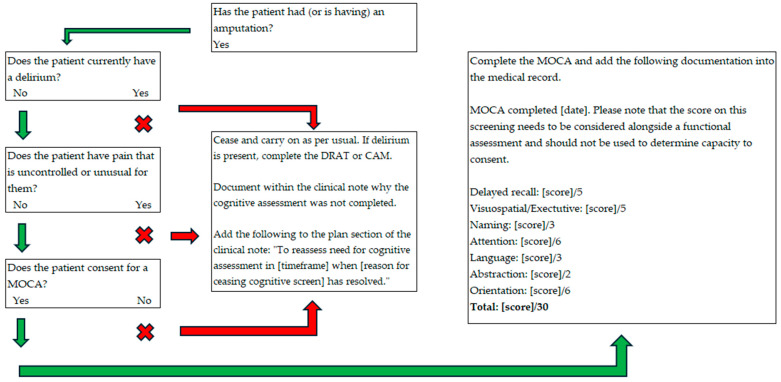
Screening Pathway for Amputee Cognition (SPArC)—inpatient pathway.

**Figure 3:**
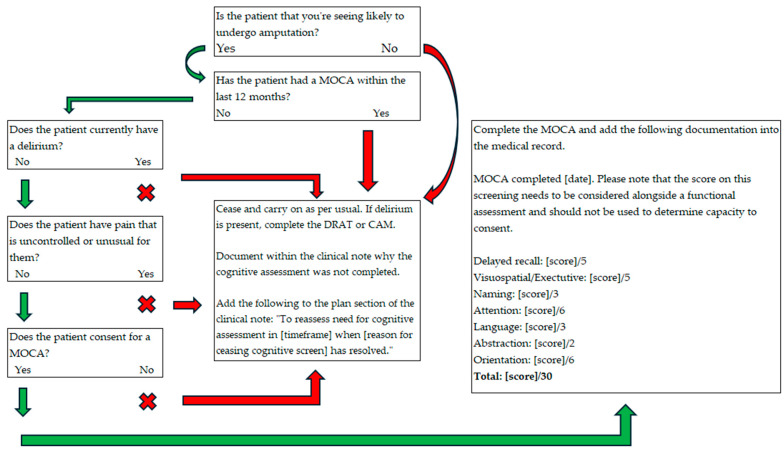
Screening Pathway for Amputee Cognition (SPArC)—outpatient pathway.

All the agreed decisions made by the codesign group were made into a pathway that can be seen in Figure 2 (inpatient pathway) and Figure 3 (outpatient pathway).

## 4. Discussion

The codesign process resulted in generation of not only an inpatient pathway for routine cognitive screening for post-operative care of a person following amputation, but the establishment of a pre-operative outpatient cognitive screening pathway that would result in patients having a registered cognitive baseline upon presentation to hospital in the event of emergency situations. The determination of which cognitive screening tool to use, as was evident in the codesign process by the amount of discussion surrounding the selection, is topical and has been extensively discussed in the literature previously. The choice of the MoCA by the codesign group as the cognitive screening tool is in line with previous research investigating the cognitive impact of vascular amputation on functional mobility outcomes [29,30].

The MoCA is reportedly one of four tools that demonstrates adequate specificity and sensitivity for MCI compared with age-appropriate cognitive controls [9]. Additionally, it is one of two identified as having the best accuracy in distinguishing VMCI from control populations [8]. This may stem from the targeted nature under which the MoCA was developed, seeking to provide a clearer boundary between itself and the MMSE [31,32]. Regardless, it is positive given that diabetes, one of the most common causes of vascular amputation, has been shown to exhibit cognitive changes that would be identified using the screening tool that has been selected. Repetition in the application, as was identified with screening occurring at two timepoints, will help to demonstrate the progression of cognitive decline if present [11]. The same authors established that a reliable accurate change is demonstrated when there is a three-point change in the unadjusted score within a 12 month period [11]. However, from a practical standpoint, others have found that a change of just one in the unadjusted score is associated with a faster ambulation speed and reduced falls risk for the amputee [33].

The eMoCA is a version of the MoCA that has been adapted to an app format, being able to be used on touchscreen phone, tablet, or laptop without formal training, under the trade name MoCA Duo [34]. It was officially launched in 2023 following testing and “allows easier administration for clinicians due to live instructions ensuring greater standardisation” [34]. The scores are automatically tabulated and a pdf is generated, presented in a similar manner to what would be expected from the paper version. Recent reports have found that there is adequate convergent validity and correlation between the paper and electronic versions of the test [35,36,37]. Some initial concerns centered around the use of a stylus and the lack of sensitivity when in contact with the test screen, which was increasing the test duration time [38]. This would only be of concern in this pathway if the length of time to complete the screen was being used as a performance outcome measure. Regardless, the stylus sensitivity issues seem to have been resolved with the formal launch, as although a stylus is used for some test items (e.g., cube copy), if the patient is not familiar and comfortable with a stylus and phone/tablet application, then they are able to complete the task on paper and have the clinician take a photo to upload into the test.

One of the benefits of the eMoCA, identified by both the platform and the literature, is the possibility to reduce clinician burden in undertaking the test [34,35,36,38]. This was certainly a consideration during this codesign process, with clinicians wishing to be able to complete cognitive screening at various timepoints through the patient’s outpatient or inpatient journey. At a practice level, for the healthcare district under discussion, the MoCA could only be administered by a trained and certified occupational therapist. Occupational therapists are not currently linked with the outpatient services of high-risk foot or podiatry clinics; thus, having a tool like the eMoCA that could be utilized by numerous clinicians was appealing in its applicability.

The addition of the caveat that the cognitive score not preclude a patient from participating in prosthetic rehabilitation has been seen in the literature before [39]. The authors noted that a cognitive score should not be taken in isolation but should give pause to clinicians prescribing rehabilitation, as cognition was found to be an independent factor for lack of improvement in outcome measures such as the Barthel Index once a patient underwent rehabilitation.

Previous literature has established that MoCA scores falling in the range of 18–25 would see the patient characterized as having MCI [29]. For these patients, considerations in the prosthetic rehabilitation process may include having increased time to attain skills and having the information presented in different manners. When MCI is considered in combination with advanced aging, we can expect an increased length of stay required to attain skills such as balance and mobility [40]. Consideration with MCI and prosthetic rehabilitation may also be given to discharge planning and whether the patient can be supported at home or needs to consider alternative living arrangements, with previous research finding that patients with higher cognitive function had increased chance of being discharged to a home location compared with a nursing home [41,42].

The success of this codesign process in establishing two cognitive screening pathways now drives the direction of future research towards one of implementation. Doing so and comparing the rate of cognitive screening from prior to the establishment of the cognitive screening pathway to after the establishment of the cognitive screening pathway will provide data on the effectiveness of the pathway with regards to meeting the recommendations for cognitive screening and thereby adhering to best practice guidelines.

### Limitations

There was no consumer involvement in the design of completion of this phase of the research, as the emphasis was on working with clinicians to determine a feasible pathway to introduce cognitive screening into the district for persons with amputations. However, it is noted that effective care and healthcare design does need to be in partnership with the consumer [43], and future research would look to include consumers/service users as part of evaluating the implementation of the developed pathway.

## 5. Conclusions

Utilizing codesign with a cohort of allied health and medicine clinicians was successful for the development of cognitive screening pathways for patients facing or having undergone amputation. The pathways developed (inpatient and outpatient) allowed ease of integration into current practice within this health district. The MOCA was found to be the most appropriate cognitive screening tool for use in this population in this particular health district, as use of the electronic version (eMOCA) allowed increased numbers of clinicians to be able to complete the cognitive screening assessment, reducing the burden on any one discipline. The applicability of the cognitive screening pathways for patients undergoing or having undergone amputation should be determined in other healthcare settings, as well as the suitability of implementing routine screening of cognition in other patient populations.

## Figures and Tables

**Table 1:** Participant inclusion and exclusion criteria.

Allied Health (Occupational Therapy, Physiotherapy, Podiatry, Psychology)	
Inclusion Registered with the governing body, Australian Health Practitioner Regulation Agency Working within the local health district	Exclusion Allied health assistants Students undertaking clinical placement hours
Medical (vascular or rehabilitation medicine)	
Inclusion Registration with the relevant governing body Three (3) or more months experience with vascular or rehabilitation medicine within the local health district Holding specialist, senior medical officer, registrar, resident medical officer, junior medical officer, visiting medical officer, or casual medical officer status	Exclusion Less than 3 months experience within the local health district Student medical officers

**Table 2:** Cognitive screening tools considered for inclusion in the pathway.

Cognitive Screen	Considerations for Use (✓/x)			Included for Use/Excluded for Use	Reason
	Executive function domain assessed?	Completion ≤ 20 min?	Readily administered by all health professionals?		
Mini Mental State Examination (MMSE)	✓	✓	✓	Excluded	Does not offer sufficient distinction for executive function
Montreal Cognitive Assessment (MoCA)	✓	✓	✓ Training recommended for paper-based test	Included	To be the primary assessment tool
Brief Memory and Executive Test (BMET)	✓	✓	✓	Excluded	Too closely resembles full neuropsychological examination which may be required later during patient journey
Addenbrooke’s Cognitive Assessment (ACE)	✓	Borderline	✓	Excluded	Lengthy assessment for clinicians to administer
Rowland Universal Dementia Assessment (RUDAS)	✓	✓	✓	Included	To be the secondary assessment tool
Oxford Cognitive Scale (OCS)	x	Borderline	✓	Excluded	Established for use in a post-stroke population—may not be appropriate for amputee population

## Data Availability

The raw data supporting the conclusions of this article will be made available by the authors on request.
